# Supplementary material for: Deletion of wfs1 Impairs Oligodendrocyte Precursor Cells Dorsal Distribution and Myelination Through the wfs1-hmgcs1 Axis in Zebrafish
Source: Biology (Basel). 2026 Aug 16;15(16):1402. doi: 10.3390/biology15161402 (PMC13510151; doi:10.3390/biology15161402)
Supplement: Supplementary file 1 [file biology-15-01402-s001.zip › File S1-Transcriptome sequencing data and differentially expressed genes (DEGs) in wfs1-- zebrafish at 5 dpf/2.KEGG/wfs1KOvsWT/ALL/src/dre03410.html]

dre03410


- dre:558198

- Down regulated genes

558198\_1(-4.91578656207803)

- dre:556995

- Up regulated genes

556995\_1(2.4821943051154)

- Down regulated genes

556995\_2(-1.79188203078216)

- dre:556995

- Up regulated genes

556995\_1(2.4821943051154)

- Down regulated genes

556995\_2(-1.79188203078216)

- dre:393774

- Up regulated genes

393774(3.89419237955922)

- Down regulated genes

393774\_2(-7.599899395667)
- dre:553405

- Down regulated genes

553405\_1(-5.15746990409678)

- dre:558198

- Down regulated genes

558198\_1(-4.91578656207803)

- dre:556995

- Up regulated genes

556995\_1(2.4821943051154)

- Down regulated genes

556995\_2(-1.79188203078216)

- dre:110438871

- Up regulated genes

110438871(1.82412391238383)

- Down regulated genes

321622(-1.53203125771205)

- dre:556995

- Up regulated genes

556995\_1(2.4821943051154)

- Down regulated genes

556995\_2(-1.79188203078216)

- dre:558198

- Down regulated genes

558198\_1(-4.91578656207803)

- dre:393774

- Up regulated genes

393774(3.89419237955922)

- Down regulated genes

393774\_2(-7.599899395667)
- dre:553405

- Down regulated genes

553405\_1(-5.15746990409678)

- dre:558198

- Down regulated genes

558198\_1(-4.91578656207803)

- dre:796446

- Down regulated genes

796446\_1(-1.34570960709418)

Close
